# Characterization of nanoemulsion of *Nigella sativa* oil and its application in ice cream

**DOI:** 10.1002/fsn3.1500

**Published:** 2020-04-21

**Authors:** Nameer Khairullah Mohammed, Belal J. Muhialdin, Anis Shobirin Meor Hussin

**Affiliations:** ^1^ Department of Food Science College of Agriculture University of Tikrit Tikrit Iraq; ^2^ Faculty of Food Science and Technology Universiti Putra Malaysia Serdang Malaysia; ^3^ Halal Products Research Institute Universiti Putra Malaysia Serdang Malaysia

**Keywords:** emulsifier, ice cream, nanoemulsion, *Nigella sativa* oil, physicochemical stability

## Abstract

The aim of this study was to develop ice‐cream product fortified with a *Nigella sativa* oil (NSO) nanoemulsion at four ratios (0% control, 3%, 5% and 10%). The NSO nanoemulsion stabilized by combinations of gum arabic, sodium caseinate, and Tween‐20 at three ratios (5%, 10%, and 15%) of emulsifiers. The results showed that 10% nanoemulsion has the highest stability and zeta potential (−31.92), and lowest change of PDI (0.182). The 5% nanoemulsion showed the lowest particle size (175.83 µm). The result demonstrated that NSO nanoemulsion improved the ice‐cream physical properties and consumer acceptability. Among the different samples, sensory evaluation revealed that ice‐cream sample of 5% nanoemulsion received more acceptability from the panelist. This results demonstrated ice cream can be fortified with NSO nanoemulsion. This means it could be used as a functional ice cream with manifold NSO health benefits.

## Graphical abstract

Nanoemulsion of *Nigella sativa* oil used as functional ingredients for ice cream. Formulation of ice cream with nanoemulsion of *Nigella sativa* oil. Characteristics of nanoemulsion and ice cream were conducted. The developed ice cream was highly acceptable by the consumers.

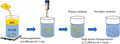

## 1 INTRODUCTION

Ice cream is a product that is consumed daily with a market value estimated at 5.75 billion liters according to USDA (2012). In addition, ice cream constitutes 86.7% of the global volume of frozen desserts (Kilara & Chandan, 2013). Ice cream is a valuable food and contains constituents that are highly nutritive for the health of humans including milk which is good sources of vitamins, proteins, and minerals (Soukoulis, Fisk, & Bohn, 2014). Recently, ice‐cream products were enriched with several bioactive ingredients to enhance their nutritional values. Several bioactive ingredients were added to ice cream such as pomegranate peel phenolics (Çam, İçyer, & Erdoğan, 2014), purple rice bran oil (Alfaro et al., 2015), whey protein (Danesh, Goudarzi, & Jooyandeh, 2017), and vitamin D3 (Tipchuwong, Chatraporn, Ngamchuachit, & Tansawat, 2017).

Modern consumers are demanding for natural foods that are rich in nutrients and may have biological functions. This demand encourages food manufacturers and researchers to introduce new formulations of ice cream that are enriched with different ingredients. However, commercial ice‐cream products are poor source of these nutrients (Sun‐Waterhouse, Edmonds, Wadhwa, & Wibisono, 2013). Several previous studies recommended to add oils that are rich in phytochemicals to enhance the nutritional value of ice‐cream products, Chia (*Salvia hispanica* L.) (Ullah, Nadeem, & Imran, 2017), hazelnut oil and olive oil (Güven, Kalender, & Taşpinar, 2018), and flaxseed oil (Gowda, Sharma, Goyal, Singh, & Arora, 2018). *Nigella sativa* oil has been suggested in food applications as functional ingredient (Mukhtar, Qureshi, & Anwar, 2019). According to Hassanien, Assiri, Alzohairy, and Oraby (2015), the biological activities of *Nigella sativa* oil are due to the presence of bioactive compounds named thymoquinone. The addition of oil into food systems is a great challenge due to the high hydrophobicity and separation of oil in the final product. Therefore, several methods were suggested for adding oil in food system such as solid dispersions, liposomes, amorphous solid form, melt extrusion, and nanocarriers.

Oil‐in‐water nanoemulsion can easily be added to dairy products to improve its nutrition value (Cheong, Tan, Tan, & Nyam, 2016). Nanoemulsions were added to several food system due to their functional properties in food processing (Anton, Gayet, Benoit, & Saulnier, 2007; Mahdi Jafari, He, & Bhandari, 2006). Sodium caseinate (NaCas) is generally used as an emulsifier in devising delivery systems to improve the stabilizing and emulsifying functions (Mohammed, Meor Hussin, & Chin Ping, 2018; Mohammed, Tan, Abd Manap, Muhialdin, & Meor Hussin, 2019; Qu & Zhong, 2017a; Shah, Ikeda, Davidson, & Zhong, 2012). To the best of our knowledge, there are no studies were carried out to determine the quality and the application of NSO nanoemulsion in ice‐cream products. Therefore, the objective of this study is to produce NSO nanoemulsion and determine the functional properties in ice‐cream production.

## 2 MATERIALS AND METHODS

### 2.1 Materials

The *Nigella sativa* L was purchased from a local supplier in Selangor state, Malaysia. Solvents (analytical grade), gum arabic, Tween‐20, and soy lecithin were purchased from Sigma‐Aldrich Co. Powder of skim milk, cream (35% milk fat) (Dutch Lady, Dutch Lady Milk Industries Bhd), were supplied by a local market in Kuala Lumpur, Malaysia. The stabilizer (guar gum) and emulsifier (sodium caseinate) were purchased from Rhodia Co, Germany.

### 2.2 Preparation of Nigella sativa oil nanoemulsion

**Figure 1:**
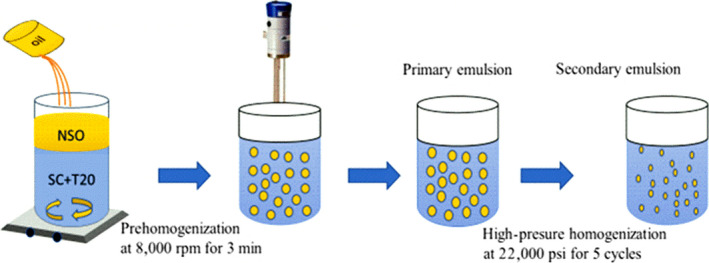
Schematic diagram of the production of *Nigella sativa* seed oil‐in‐water nanoemulsion

The aqueous phase contains accurate measures of sodium caseinate (SC) (64.9 w/w), gum arabic (GA) (6.4 w/w), and Tween‐20 (T20) (28.7% w/w), as the optimum proportions for oil nanoemulsion reported by Cheong et al. (2016). Three total emulsifier concentrations of the emulsifier mixture (5%, 10%, and 15%) were carefully prepared to assess the ideal amount in percentages of the aqueous phase for the NSO nanoemulsion preparation. The extraction of *Nigella sativa* oil (NSO) was done by supercritical fluid extraction (SFE) followed the approach of Mohammed et al. (2016). The NSO nanoemulsions were prepared as shown in Figure 1. Using a hot plate with a magnetic stirrer into the three aqueous phases which contained blends of emulsifier, NSO was added dropwise at 50°C (Figure 1). Then, the three coarse emulsions which were coded as A5, A10, and A15 were prepared at aqueous phase to oil ratio of 10:90% (w/w) which were fixed. First, coarse emulsion A5 was prepared with 5% aqueous phase + 10% oil + 85% water; second, A10 was prepared with 10% aqueous phase + 10% oil + 80% water; and finally, A15 was prepared with 15% aqueous phase + 10% oil + 75% water. The emulsions were stirred for 10 min for the formation of coarse emulsion. The oil‐in‐water coarse emulsions were formed at room temperature (25 ± 2°C) prehomogenized at 7,168 *g* for 3 min, in a mixer of the high shear (Ultra‐Turrax, IKA UK). Nanoemulsion was prepared using a high‐pressure homogenizer (Nano De BEE, BEE International) as reported by (Cheong et al., 2016). The primary emulsions were homogenized at 151 MPa for five cycles. The cycle and pressure of homogenization in this study were kept constant.

### 2.3 Characterization of nanoemulsion

#### 2.3.1 Polydispersity index and particle size

The measurement of PDI and z‐averages (mean particle size) was carried out using ultrapure water (1:10) to evade the effects of multiple scattering and adopting Zeta sizer Nano‐ZS (Malvern Instruments Ltd.; Anarjan & Tan, 2013). The samples were diluted before conducting the measurement and refractive indexes for temperature and water were set at 25°C and 1.33. The three measurements’ averages and standard deviations were recorded. The size droplets distribution width is represented in the PDI, in which a 0.1–0.25 PDI value means a narrow size distribution according to Patravale et al. (2004), while a value > 0.5 shows a particle size broad distribution according to Gibis, Rahn, and Weiss (2013).

#### 2.3.2 Zeta potential

The zeta‐potential values was measured directly, to determine the particle charge of nanoemulsion using capillary cuvette DTS 1,060 was equipped with electrode after dilution with ultrapure water (1:10), (Malvern Instruments Ltd.). In this procedure, potential value of zeta gives a pointer of the stability of the nanoemulsion through determining the electrostatic magnitude, and the repulsion or attraction charge between particles. An emulsion that is stable must attain 30 mV (positive or negative) at least, of zeta‐potential value, in order to sustain stability, as in this case, it is the electrostatic repulsions minimum between particles, which is sufficient to stop them from coalescence (Guterres, Poletto, & Colomé, 2010).

#### 2.3.3 Creaming test

After homogenization, 10 ml of nanoemulsions was straightaway transferred into a test tube, sealed tightly and stored for 7 days, at room temperature (25 ± 2°C). The stability of the creaming was measured through observing visually and subsequently measured by creaming index percent = (*H*_L_/*H*_E_) × 100%, in which *H*_E_ = the overall height of emulsions, and *H*_L_ = the total height of the layer of the cream (Surh, Decker, & McClements, 2006). High creaming index points to emulsion instability which occurs because of coaleascence, aggregation, flocculation, or large particle size (Bouyer, Mekhloufi, Rosilio, & Grossiord, 2012).

#### 2.3.4 Creaming stability

The creaming was determined through observing visually, then measured by creaming index percent = (cream layer height/total emulsions height) × 100 (Surh et al., 2006).

#### 2.3.5 Morphology of nanoemulsion

Transmission electron microscopy (TEM) was used to assess the structure and morphology of nanoemulsion. In distilled water (1/10), the formulation of nanoemulsion was diluted; then, the diluted nanoemulsion drop was placed on film grid of holey and was observed subsequently after drying. Samples were subsequently taken out and were tested under a TOPCON 002B operating on 200 kV, which has the potential of point‐to‐point resolution, for it to perform the TEM observations. At increasing magnification, a blend of bright field diffraction modes imaging was utilized to reveal the nanoemulsion form and size.

### 2.4 Ice‐cream preparation

**Figure 2:**
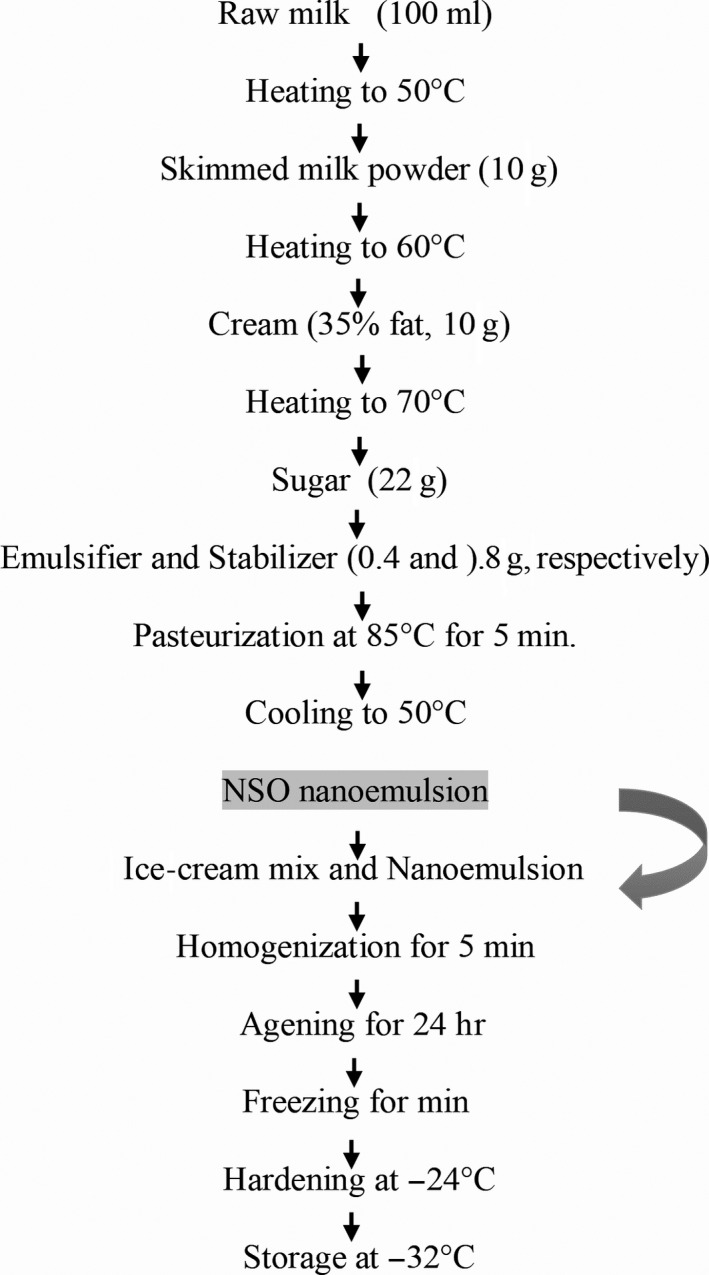
Flowchart of the ice‐cream production with NSO nanoemulsion

For succeeding ice‐cream fortification, the NSO nanoemulsion prepared with 10% emulsifier ratio, namely, A10 formulation (10% oil, 10% emulsifier, 80% deionized water) was used. According to Al‐Ali, Alkhawajah, Randhawa, and Shaikh (2008), the doses of TQ which are recommended for antioxidant, anti‐inflammatory, and anticancer effects are 5–10 mg/kg. According to a previous study by Mohammed et al. (2016), 1 ml of NSO extracted through SFE has a 6.63 mg thymoquinone content and antioxidant activity which is expressed as IC_50_ 1.58 mg/ml of oil. Therefore, the ice cream was produced with the minimum allowed dose of NSO. Based on calculations, 10% of nanoemulsion gives a 6.6 mg of TQ, which is within the limit for the daily human oral doses as Tisserand and Young (2013) reported, an amount of thymoquinone which is between 5 and 90 mg/kg has the potential of being therapeutic and nontoxic of thymoquinone. Ice‐cream samples which were supplemented with NSO nanoemulsion were prepared according to the process flowchart in (Figure 2). On dry matter bases, four diverse samples of ice cream are formulated on 0%, 3%, 5%, and 10% of NSO nanoemulsion and coded as T0, T1, T2, and T3, respectively. The first step involved the heating of milk to 50°C, followed by adding the solid ingredients (sugar, cream, skim milk powder, and emulsifier or stabilizers were incorporated at 60°C). The pasteurization of the mixture took place for 5 min at 85–90°C and subsequently cooled rapidly to 50°C. Nanoemulsion of NSO was then added at this stage (after pasteurizing the mixture), with continuous mixing, and the mixture was subjected to a homogenization at 4,032 *g*, then kept at 4ºC overnight, for the aging of ice‐cream mix. After aging process, using an ice‐cream maker, the samples were allowed to speedily harden at −24°C (Simac II Gelataio GC 6,000; Simac). This was followed by the collection of the ice‐cream samples in 0.5 kg plastic containers, and hardening it at −20°C for 48 hr.

#### 2.4.1 Overrun

The ice‐cream samples (the three batches) were tested for their melting point and overrun following the method described in previous study (Arbuckle, 1986; Schmidt, 2004). The calculation of overrun was done with the following equation “% Overrun = (Vol. of ice cream − Vol. of mix used)/Vol. of mix used × 100%.”

$$\text{Overrun}=\frac{\text{Wt}\text{. of icecream mix - wt}\text{. of icecream}}{\text{Wt}\text{. of icecream}}\times 100$$

#### 2.4.2 Melting resistance

The assessment for the behavior of melting was carried out by collecting of ice‐cream containers (120 g) at −20°C from the freezer to determine the meltdown point. The samples were placed on rectangular screens of stainless‐steel wire mesh (1.5–1.5 cm, whole size of 2.5–2.5 mm) atop of a funnel which is connected to a graduated cylinder. Then, at −25°C, the ice cream was sited in a chamber with a temperature that is controlled. For up to 90 min, every 10 min, the dripped volume was weighed and recorded. The recording of the times of the first drop of ice cream trickled and the time at which complete melting happened were recorded. At 30‐min intervals, the bulk of unmelted ice cream was recorded using graduated cylinder. The time was plotted against the trickled volume (ml), while the slope of the core melting event was considered as the rate of melting.

#### 2.4.3 pH values and total soluble solid content

The total solid content which is soluble was measured at −20°C in triplicate using a refractometer (Pocket Pal‐1, Atago, Tokyo, Japan) and expressed as °Brix. The determination of pH was done using pH meter (850Schott Instruments, Germany).

#### 2.4.4 Texture analysis

The texture analysis was performed by storing the ice‐cream samples in plastic containers at −20°C for 24 hr. Firmness, hardness, consistency, cohesiveness, and viscosity index determinations were taken at room temperature (25 ± 2°C), using texture analyzer TA‐XT2i (Stable Microsystems Ltd., UK) equipped with a 2‐mm‐diameter acrylic cylindrical probe. Penetration depth of the samples' geometrical centers was 10 mm, and the speeds of penetration were set at 1 mm/s. Hardened at −30°C, the ice cream was cut to fill a small cup which is cylindrical (4.5 cm diameter) to a 30 mm depth, and overnight tempered to −15°C before the analysis. The probe's penetration speed was 2 mm/s to a distance of 20 mm.

### 2.5 Sensory evaluation

The ice‐cream samples were subjected to sensory analysis following the method described by Çam et al. (2014), with some modifications. Consisting of 30 members, panelists were graduate students or academics who are 19–47 years old (17 females, 13 males) in the food science and technology faculty, University Putra Malaysia (UPM). Four samples of ice cream are formulated on 0%, 3%, 5%, and 10% of NSO nanoemulsion, namely, T0, T1, T2, and T3, respectively, were evaluated for taste, preference, appearance, and consistency, while adopting the 9‐point hedonic scale, where the score 1 indicated a very low preference, and 9 means a very high preference.

### 2.6 Statistical analysis

All analyses and formulations were performed at triplicate, and the data were presented as a mean of each experiment. Tukey's test was applied for post hoc multiple comparisons of means ± standard deviation (*SD*), and (*p* < .05) was considered statistically significant. For each enriched or control ice cream, experimental replication included three independent batches. For optimal results, a minimum of three observations per analysis were conducted. The analysis of data was carried out with the use of one‐way ANOVA by Minitab 16 statistical package (Minitab Inc.).

## 3 RESULTS AND DISCUSSION

### 3.1 Nanoemulsion characterization

#### 3.1.1 Zeta potential

The extent of electrical repulsion force between particles was measured by Zeta potential, and it was engaged to ascertain the stability of the nanoemulsions. Zeta potential of three nanoemulsion samples differs in their concentrations of emulsifiers including A5, A10, and A15 were recorded as (−26.35, −31.92, and −14.17), respectively (Table 1). A high value of zeta potential theoretically shows high stability, while low value of zeta potential means a low stability as repulsive force between the droplets that is low prevents them from flocculate and close contact (Bouyer et al., 2012). The adequate droplets electrostatic repulsion for nanoemulsion to prevent coalescence should be maintained at zeta‐potential value of ±30 mV (Yin, Chu, Kobayashi, & Nakajima, 2009). The results obtained in this study showed significant differences (*p* < .05) among the samples suggesting that emulsifier concentrations had effect on the NSO nanoemulsions stability. Thus, the A10 sample exhibited the highest value of zeta potential indicating highest force of electrostatic repulsion between droplets which resulted in a stable emulsion. Conversely, A5 and A15 samples failed to meet the minimum requirement (±30 mv) having values −26.35 and −14.17%, respectively, showing low stability of the nanoemulsion.

**Table 1:** Particle size, zeta potential, and PDI of fresh and reconstituted *Nigella sativa* oil nanoemulsions (5%, 10%, 15%, and 0% control)

Characteristics	A5%^a^	A10%^b^	A15%^c^
Mean particle size (nm)	175.83 ± 1.54^a^	188.4 ± 1.36^b^	670.06 ± 1.67^c^
PDI	0.293 ± 0.014^b^	0.182 ± 0.013^c^	0.415 ± 0.023^a^
Zeta potential (mV)	−26.35 ± 1.7^b^	−31.92 ± 2.0^a^	−14.17 ± 1.2^c^
Creaming index (%) day 0	0 ± 0.0^a^	0 ± 0.0^a^	0 ± 0.0^a^
day 7	32 ± 0.2^a^	0 ± 0.0^c^	1.7 ± 0.3^b^
Viscosity (cP)	12.3 ± 0.4^c^	19.2 ± 0.3^b^	27.3 ± 0.5^a^

Abbreviation: PDI, polydispersity index.

Values mentioned above are mean ± *SEM* (*n* = 3). Mean values within a row with unlike superscript letters were differed significantly (*p* < .05).

^a^ Combinations of gum arabic, sodium caseinate, and Tween‐20 (5%).

^b^ Combinations of gum arabic, sodium caseinate, and Tween‐20 (10%).

^c^ Combinations of gum arabic, sodium caseinate, and Tween‐20 (15%).

The result obtained in this study might be substantially due to insufficient repulsive forces surrounding the droplets of oil which contributes to the stabilization of electrostatic. This is occasioned by the emulsifiers that act to prevent the coalescence of droplets and combine them (Tadros, 2013). As a consequence of gravitational force exerting action on the diverse aqueous phase and density of oil, coalescence inducing increase in particle size could be destabilized further to sediment (McClements, 2005).

#### 3.1.2 Polydispersity index (PDI) and particle size

The development of formulation is an important factor in the production of nanoemulsion to obtain desired criteria of a small particle size (nanometer range: 20–200 nm diameter) and low PDI (<0.5). In addition, the particle size is crucial because this parameter is responsible for assessing the stability, appearance, bioavailability, and the end‐product texture (Acosta, 2009; McClements, Decker, & Weiss, 2007). Nanoemulsions were produced using three different formulations including A5, A10, and A15 showed significant differences (*p* < .05) among their particle sizes (175.83, 188.4, and 670.06 nm, respectively; Table 1). Sample A5 showed the smallest particle size (175.83 nm) among the 3 nanoemulsions, but having wide size distribution, as shown from the results of PDI (0.293). Furthermore, A10 showed similar particle size compared to A5, but having narrow distribution of particle size, which is indicated by the values of PDI (0.182). This can because the particles present in the nanoemulsion were covered by the emulsifiers, and subsequently, higher concentration might render undesired particle size. The obtained results were in agreement with previous study, indicated that β‐carotene nanoemulsion produced with high concentrations of emulsifiers showed larger particle size Yuan and Gao (2008). Increasing the emulsifier concentration from 5% to 15% (w/w) resulted in distribution of particle size to have plateau value. The findings agree with the study reported by Frascareli, Silva, Tonon, and Hubinger (2012) that indicated the increase of solid concentration (10%–30%) in the emulsion of coffee oil resulted in an increase in mean diameter of particle size (7.88–13.13 µm).

In addition, the distribution of particle size in the three nanoemulsions did not show consistent trends. The nanoemulsion showed the widest range of particle size distribution at highest emulsifier concentration (A15) followed by A5 and A10 samples (Table 1). The value of polydispersity indexes (PDI) measures the spread of the distribution of the particle size, and a small PDI value refers to a narrow distribution of particle size (Lemarchand, Couvreur, & Vauthier, 2003). In this study, three nanoemulsion samples (A5, A10 and A15) demonstrated significant differences (*p* < .05) in their PDI values 0.293, 0.182, and 0.415, respectively. PDI < 0.25 shows a very narrow particle size distribution, while PDI > 0.5 indicates particle size distribution that is large (Gibis et al., 2013). A10 showed the smallest (PDI = 0.182) among all the nanoemulsions, and this is likely to have higher physical stability. The results of PDI correlated with the particle size and indicated that the concentration of emulsifier will have significant effect on nanoemulsion stability.

#### 3.1.3 Creaming test

**Figure 3:**
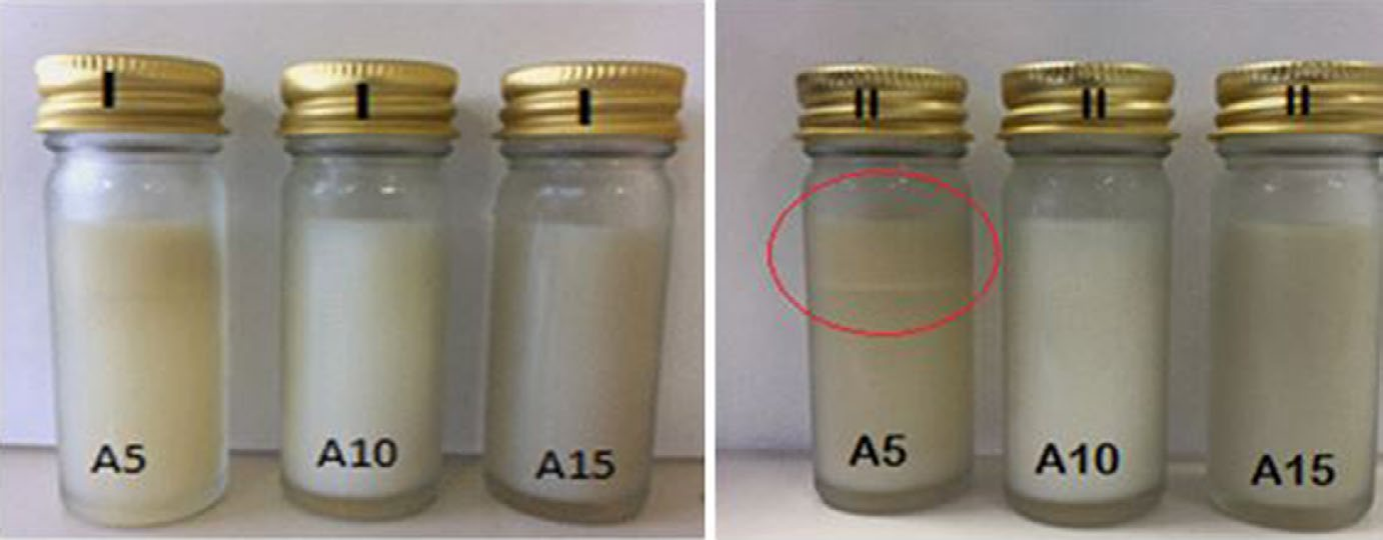
Optical photographs of the NSO nanoemulsions prepared using combinations of gum arabic, sodium caseinate, and Tween‐20 emulsifier A5, with 5% emulsifiers, A10, with 10% emulsifiers, and A15, with 15% emulsifiers. (I) Fresh emulsions; (II) the emulsions after storage for 7 days at 25°C

Optical photographs were snapped for the fresh and the 7 days stored NSO nanoemulsions to visually observe the creaming index (Figure 3). The different formulations of NSO nanoemulsion including A5, A10, and A15 showed significant differences (*p* < .05) for their indexes of creaming stability. The nanoemulsion A10 produced with 10% emulsifiers, indicated more stability, and at the same time, after storage for 7 days no creaming formation was observed. In comparison, A5 and A15 nanoemulsions samples formulated with 5% and 15% emulsifiers, respectively, revealed significant differences (*p* < .05) and with less stability after the storage time. The results of the creaming stability correlated well with zeta potential as sediment or creaming was not observed with zeta‐potential value greater than −30 mV for nanoemulsions.

The A15 sample prepared with the highest concentration of emulsifiers indicated no stability over the storage time. This can be the effect of using a high SC concentration due to the nanoemulsion undergoing of caseinate‐coated droplets depletion flocculation caused by nonabsorbed caseinate of submicelles (Huck‐Iriart, Álvarez‐Cerimedo, Candal, & Herrera, 2011). In addition, A5 sample prepared with 5% emulsifier concentration was also recorded to have low stability in comparison with sample A10 (Figure 3). Cheong et al. (2016) reported similar results for the low emulsifiers 5% (w/w) concentration which was not enough to adequately encapsulate the droplets of oil and resulted in the destabilization. Figure 3 showed that sample A10 had no formation of cream after 7 day storage at 25°C. Hence, in order to attain nanoemulsions which are most stable in terms of creaming, adequate concentration which is near the value of saturation surface coverage must be achieved. The process provides an amount of protein that is sufficient in order to promote surface coverage that is excellent, and at the same time, it relates also to stabilization of electrostatic toward coalescence and creaming (Huck‐Iriart et al., 2011).

#### 3.1.4 Viscosity determination

According to Lovelyn and Attama (2011), viscosity is a very crucial determining factor of stability and efficient drug release. The viscosities of the three NSO nanoemulsion samples including A5, A10, and A15 prepared with different emulsifier concentrations had a different values 12.3–19.2–27.3 cP, respectively (Table 1). The viscosity of nanoemulsions is a function of the surfactant, oil, water, and components of emulsifiers. Sample A5 having the least viscosity in all the nanoemulsion samples (12.3 cP) which is significantly different (*p* < .05) because of its lower concentration of aqueous phase. The low viscosity can be as a consequence of high water content coupled with low concentration of emulsifiers. Low emulsifier concentration will lower the interfacial tension between water and oil and decreased the viscosity (Lovelyn & Attama, 2011). Polysaccharide‐rides like gum arabic improve stability by continuous phase increasing viscosity and forming 3‐dimensional networks to confine the droplet movement and reduce coalescence and droplet creaming (Adjonu, Doran, Torley, & Agboola, 2014).

#### 3.1.5 Morphology of nanoemulsion

**Figure 4:**
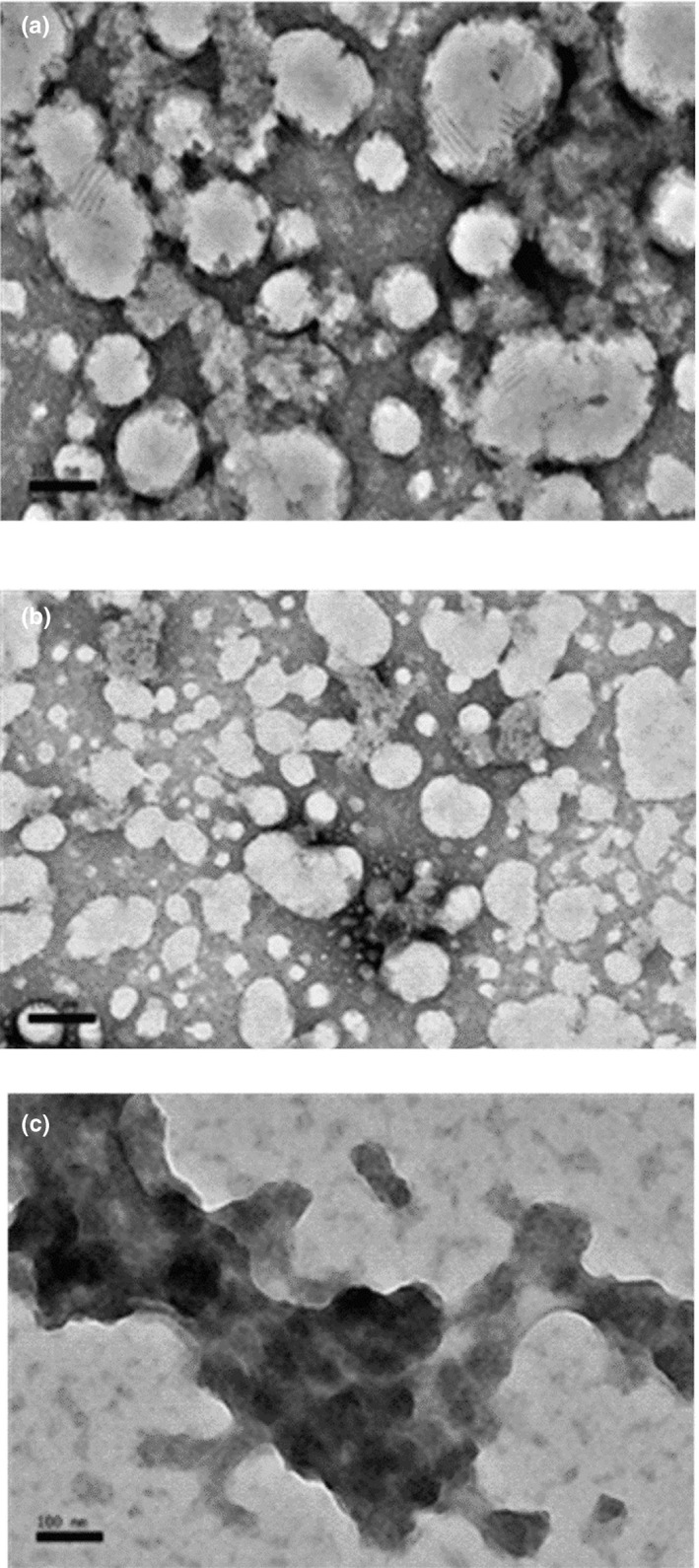
Transmission electron micrographs of NSO nanoemulsions (a) prepared with 5% emulsifiers; (b) with 10% emulsifiers; and (c) with 15% emulsifiers

Transmission electron microscopy (TEM) was immediately applied after the nanoemulsion formulations produced to visualize the morphology of the particles. TEM was performed to confirm the particle size and internally assure the perfect encapsulation of NSO within the three concentration of coating materials of 5% (A5), 10% (A10), and 15% (A15) (Figure 4). The samples manifested particle sizes more than 100 nm diameter, and this was correlated with the particle size. Samples A5 and A10 particles morphology were spherical with slight differences.

Analogous results were reported by Sharma et al. (2017) that the TEM image showed the nanoparticle of clove oil nanoemulsion had a spherical shape with a size of approximately 200 nm.

On the other hand, the morphological graph of A15 sample appeared dark, and the surroundings are bright indicating spindle‐shaped irregular shape. This might be due to the collapse of oil droplet when the nanoemulsion deposited on TEM copper grid coated with a holey carbon film (Ding et al., 2013). Compared with samples A5 and A10, the size of sample A15 was much bigger with an irregular shape, mostly due to smaller particles agglomeration. Yuan, Gao, Zhao, and Mao (2008) reported similar results for sunflower oil nanoemulsion with ß‐carotene which was prepared with diverse concentrations of Tween‐20 emulsifier and demonstrated spherical shapes with different sizes.

### 3.2 Characterization of ice cream

#### 3.2.1 Melting resistance

Melting resistance happens is one of the most crucial physical properties of ice‐cream products. In this study, ice‐cream melting resistance was improved with NSO nanoemulsion (A10) because of its high stability at different concentrations (Table 2). Four samples of ice cream were formulated with four different concentrations of NSO nanoemulsion: 0% (T0), 3% (T1), 5% (T2), and 10% (T3). The melting rates order of the ice‐cream mixture was in the sequence that follows: T3 < T2 < T0 < T1 having showed significant differences (*p* < .05) as shown in Table 2. The T3 formulation was observed to be melted after 153 min, while after 148 min, the T2 ice‐cream formulation was melted. The T1 ice‐cream formulation began melting 2–3 min earlier than the other three samples of ice cream, and accordingly, its melting rate happens to be the greatest especially after 100 min. When high nanoemulsion percentage was used for the formulation of ice cream, high‐melting resistance was observed. This is due to the functional properties of the ingredients used for *Nigella sativa* oil nanoemulsion. According to Mohammed et al. (2019), a combination of sodium caseinate, maltodextrin, and soy lecithin was successfully encapsulated of NSO and used as a nondairy creamer with desirable properties.

**Table 2:** Overrun, Milting resistance, pH, and Total solids of *Nigella sativa* oil nanoemulsions

Samples	Overrun (%)	Milting resistance (g/min)	pH	Total solids
T_0_	66.7 ± 2.2^c^	143 ± 1.4^c^	6.94 ± 1.8^a^	15.3 ± 1.2^b^
T_1_	66.7 ± 2.1^c^	137 ± 1.5d	6.75 ± 2.4^b^	15.8 ± 1.4^b^
T_2_	72.4 ± 1.74^ab^	148 ± 2.7^b^	6.62 ± 1.6^bc^	16.4 ± 1.7^ab^
T_3_	75.4 ± 2.14^a^	153 ± 0.9^a^	6.53 ± 1.1^bc^	17.4 ± 0.6^a^

Note

Values mentioned above are mean ± *SEM* (*n* = 3). Mean values within a row with unlike superscript letters were differed significantly (*p* < .05).

T0, T1, T2, and T3 are ice‐cream formulations (control) containing 0% NSO nanoemulsion, 3% NSO nanoemulsion, 5% NSO nanoemulsion, and 10% NSO nanoemulsion, respectively.

Sodium caseinate (NaCas) is mostly used in fabricating delivery systems as an emulsifier. Qu and Zhong (2017b) reported that when proteins are conjugated by reducing saccharides such as maltodextrins (MD), it can increase the emulsification and stabilization functions of oil/water (O/W) emulsions. Zhang et al. (2017) reported the oil‐in‐water emulsions freeze–thaw stability with sodium caseinate–maltodextrin conjugates which brought about a substantial improvement in freeze–thaw stability as compared with the mixture of control protein and protein–sugar. In addition, Granger, Leger, Barey, Langendorff, and Cansell (2005) reported the preparation of the ice cream which contains refined and hydrogenated coconut oils and refined palm oil that in combination with the saturated monoglycerides and diglycerides emulsifier. The researcher observed that the melting times depended on the ice‐cream formulation and the nature of the emulsifier with less effects of the oil type. It is highly not expectable that there may be any observed discrepancy in the time of the first drip of the samples, though there is an observed significant difference between the rates of melting, once the structure commences deforming (Kumar et al., 2016). Nazaruddin, Syaliza, and Wan Rosnani (2008) stated that the air presence in ice creams has effect on its melting characteristics. As an example, the ice‐cream overrun volume is increased. Higher overrun gives out higher and softer ice creams melting percentage. Therefore, this study observes that the greater melting resistance of the T2 and T3 ice cream is linked with the highest overrun (72 and 75 g/100 g), which approves this suggestion.

#### 3.2.2 Overrun

The maximum overrun matches with the maximum stiffness and stability of the foam (Jakubczyk & Niranjan, 2006). According to Marshall, Goff, and Hartel (2012), overrun of ice creams is the measurement for the air content that form of cells or microscopic bubbles which required to be appropriately formed and stabilized during manufacturing. The values of overrun of the ice cream that is formulated with *Nigella sativa* oil nanoemulsions are shown in Table 2. The overrun value of T3 (ice cream formulated with 10% of NSO nanoemulsion) observed to be higher compared to the control and T1 sample. High percentage of overrun shows high stability and stiffness of the foam of the formulation of ice cream. The sodium caseinate formulation and maltodextrin increased the overrun value for (66.7% to 75.4%), due to the functional properties as stabilizing and emulsifying agents. NSO nanoemulsion was an extraemulsifier and stabilizer agent which improved the properties of final product. However, the overrun values for the four formulations showed lower overrun values compared with the results reported by Borrin, Georges, and Brito‐Oliveira (2018), were in the range of (129% to 144%) for the ice cream incorporated with curcumin‐loaded nanoemulsion. On the other hand, it is higher than values reported by Alfaro et al. (2015), and rice bran oil nanoemulsion containing purple had overruns between (19%–22%).

#### 3.2.3 pH values and total soluble solid content

The samples of ice cream T0, T1, T2, and T3 soluble solid contents were 15.3, 15.8, 16.4, and 17.4 °Brix, respectively (Table 2). The ice‐cream sample T3 found to have significantly greater (*p* < .05) total solid soluble content than other samples. With NSO nanoemulsion addition, the samples pH value was significantly (*p* < .05) changed. The highest pH value observed was 6.53 for the (T0) control sample, without nanoemulsion addition. Adding 3% of NSO nanoemulsion (T1) changed the pH value to 6.75, (T2) formulated with 5% NSO nanoemulsion changed the pH value to 6.62, and (T3) formulated with 10% NSO nanoemulsion changed the pH value to 6.53. The lowest pH value found was in sample T3, having the highest percentage of nanoemulsion, indicating higher acidity. In general, addition of NSO nanoemulsion resulted in reducing the pH values, substantially due to the oil's high content of phenolic.

In previous study, Sagdic, Ozturk, Cankurt, and Tornuk (2012) reported that the addition of different phenolic substances like gallic acid, ellagic acid, and extracts from peppermint and grape seed with substance increased the acidity of ice‐cream sample (*p* < .05). In another study, Hwang, Shyu, and Hsu (2009) observed that when grape wine rich in phenolic substance is added to the ice cream, it induces a pH values due to acidic substance. Conversely, investigating special herb prospects, saffron's addition to the ice cream for the improvement of the product acceptability, Çelik, Cankurt, and Doğan (2010) observed that the saffron induced pH significant increase.

#### 3.2.4 Texture analysis

The organoleptic properties and consumer acceptability of the product are highly affected by the consistency and viscosity of the product. The NSO nanoemulsion amount in the ice‐cream samples mixtures showed changes their rheological properties. The values were significantly influenced by the added percentage of NSO nanoemulsion (Table 3). Sample (T3) formulated with 10% NSO nanoemulsion gave the highest values for texture characteristics including cohesiveness (0.429 g), firmness (251.016 g), index of viscosity (25.789 gs), and consistency (5.480 gs). The control sample without addition of NSO nanoemulsion showed no significant differences compared to T2 sample that contains 5% nanoemulsion. The results indicated that 5% of nanoemulsion added has no significant alter to the standard texture properties, stability, and stiffness of the ice cream. Guo et al. (2018) reported that the textural profile of the ice cream was not affected by the nanobacterial cellulose complex gel when added at low percentage to the ice cream as fat substitutes. The firmness values ranged from 176.58 to 251.01 (Table 3). These values had significantly (*p* < .05) affected by the addition of the nanoemulsion, and the highest value observed for T3 ice‐cream sample containing 10% nanoemulsion. The firmness values obtained were in accordance with the study reported by McGhee, Jones, and Park (2015), and firmness values were varied from 182.8 to 297.3 for the ice cream produced with different types of goat milk.

**Table 3:** Rheological properties of *Nigella sativa* oil nanoemulsion

Parameters	T0	T1	T2	T3
Firmness (±*SD*) (g)	194.66 ± 1.06^b^	176.58 ± 0.38^a^	198.13 ± 1.68^b^	251.016 ± 0.24^d^
Consistency (±*SD*) (gs)	9.844 ± 0.48^a^	8.883 ± 0.99^b^	9.018 ± 0.46^a^	5.480 ± 0.48^c^
Cohesiveness (±*SD*) (g)	0.254 ± 0.004^c^	0.362 ± 0.36^ab^	0.351 ± 0.59^ab^	0.429 ± 0.09^a^
Index of viscosity/consistency (±*SD*) (gs)	24.046 ± 2.48^a^	23.100 ± 2.48^a^	23.868 ± 1.48^a^	25.789 ± 2.48^a^

Note

Values mentioned above are mean ± *SEM* (*n* = 3). Mean values within a row with unlike superscript letters were differed significantly (*p* < .05).

T0, T1, T2, and T3 are ice‐cream formulations (control) containing 0% NSO nanoemulsion, 3% NSO nanoemulsion, 5% NSO nanoemulsion, and 10% NSO nanoemulsion, respectively.

### 3.3 Sensory evaluation

**Figure 5:**
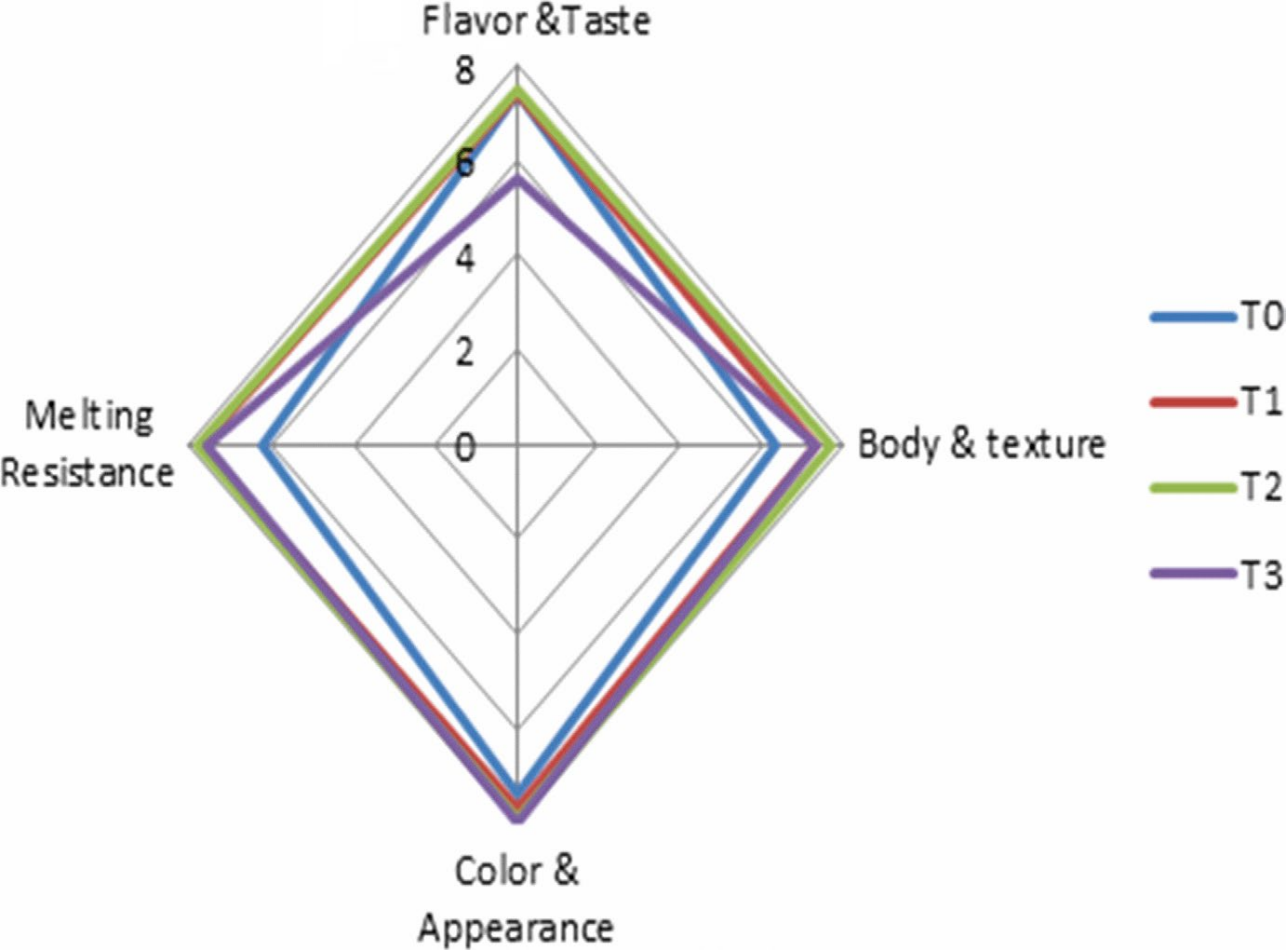
Sensory evaluation scores of enriched ice‐cream formulations (T0) control, (T1) = 3% nanoemulsion of NSO, T2 = 5% nanoemulsion of NSO, T3 = 10% nanoemulsion of NSO

The sensory evaluation was carried out using nine points Hedonic Scale from extremely dislike (1) to extremely like (9) (Goudarzi, Madadlou, Mousavi, & Emam‐Djomeh, 2015). Based on panelists’ perceptions, a taste was rated as acceptable for the ice cream (T1) formulated with 3% NSO nanoemulsion, and ice cream (T2) formulated with 5% NSO nanoemulsion indicated no negative effect on the taste scores. However, when the concentration of NSO nanoemulsion is increased up to 10% (T3), it resulted in lower taste scores. Cheikh‐Rouhou et al. (2007) reported that *Nigella sativa* oil has a bitter or strong peppery taste in the mouth and may have effects on the food products. High NSO concentration has a bitter taste, which is likely to have effect on the consumer's perceptions. The result of this study agrees with the findings of Abedi, Rismanchi, and Shahdoostkhany (2016) reported that yogurt enriched with microencapsulated NSO has a bitterness for the high content due to the NSO nature. On the other hand, no significant differences were observed the color between the control and NSO enriched samples. It can be observed that NSO nanoemulsions have white colors with high brightness and indicated no effect on the color of ice‐cream samples (Figure 5). The values of body and texture for all samples containing NSO nanoemulsions give higher scores compared to that of T0 control. Furthermore, the mixture of maltodextrin with water resulted in a smooth gel‐like matrix which can be used instead of fat. This produces acceptable flow properties and lubricant by improving the viscosity and producing a creamy and smooth mouth feel, akin to fats.

## 4 CONCLUSION

This study elucidated that NSO can be produced successfully in a nanoemulsion form. The characterization for the typical NSO nanoemulsion resulted in acceptable particle size, stability, and morphology. The results demonstrated that the addition of NSO nanoemulsion to ice cream enhanced the rheological and sensory properties. The ice‐cream samples sensory evaluation revealed that ice cream (T2) formulated with 5% nanoemulsion of NSO was the most preferred among the four formulations. It can be concluded that NSO in a nanoemulsion form has the potential to be utilized in the ice‐cream industries. Further studies should be conducted to measure *Nigella sativa* oil release in gastrointestinal conditions, which are simulated to evaluate in detail this encapsulation technique.

## CONFLICT OF INTEREST

The authors declare that they do not have any conflict of interest.

## ETHICAL APPROVAL

The study did not involve any human or animal testing.
